# Investigating the Impact of hsa_circ_0005255 on Proliferation and Autophagy in Crohn's Disease Intestinal Epithelial Cells Through miR‐23a‐3p‐Mediated NCOA3 Expression

**DOI:** 10.1002/kjm2.70035

**Published:** 2025-05-07

**Authors:** Dong Liu, De‐Run Kong

**Affiliations:** ^1^ Department of Gastroenterology The First Affiliated Hospital of Anhui Medical University Hefei City China; ^2^ Department of Gastroenterology The Third People's Hospital of Hefei and Hefei Third Clinical College of Anhui Medical University Hefei City China

**Keywords:** autophagy, Crohn's disease, hsa_circ_0005255, miR‐23a‐3p, NCOA3

## Abstract

Crohn's Disease (CD), an inflammatory bowel disorder, is influenced by genetic, immune, and environmental factors. The present study highlights the pioneering role of circular RNAs (circRNAs) in the etiology of CD, with a specific focus on hsa_circ_0005255 and its regulatory role. Utilizing both bioinformatic and experimental approaches, we exposed the mechanistic and therapeutic significance of hsa_circ_0005255 within the pathophysiological framework of CD. Our findings revealed a significant underexpression of hsa_circ_0005255 in tissue samples from CD patients and in DSS‐induced CD mouse models. The overexpression of hsa_circ_0005255 markedly mitigated inflammatory responses, as indicated by decreased serum levels of tumor necrosis factor‐alpha, interleukin‐1 beta, and interleukin‐6, and reduced histopathological indications of inflammation in colonic tissues. It substantially improved the integrity of the epithelial barrier, evidenced by the upregulation of Zonula Occludens‐1 expression and the reduction of apoptosis in colonic epithelial cells. Furthermore, this regulatory effect extended to the enhancement of epithelial cell proliferation and autophagy, characterized by the elevated expression of Ki‐67, microtubule‐associated protein 1A/1B‐light chain 3 II, and Beclin‐1, along with the suppression of cleaved caspase‐3 and sequestosome 1. Mechanistically, hsa_circ_0005255 functioned as a competitive endogenous RNA, absorbing miR‐23a‐3p and thereby regulating Nuclear Receptor Coactivator 3. This investigation not only broadens our understanding of the involvement of circRNAs in CD pathogenesis but also identifies hsa_circ_0005255 as a potent biomarker and therapeutic target.

## 1 Introduction

A chronic inflammatory bowel disorder (IBD), Crohn's Disease (CD) can affect any area of the gastrointestinal tract, ranging from the oral cavity to the anus. The condition involves periodic inflammatory episodes that can penetrate through all layers of the intestinal wall [1, 2]. The etiology of CD remains incompletely elucidated; however, it is generally accepted that it results from a mixture of genetic predispositions, aberrant immune responses, and environmental factors [3]. At the heart of CD pathogenesis lies an inflammatory response, where an exaggerated immune reaction against the normal gut microbiota or unidentified antigens results in persistent inflammation [4]. The paramount objectives in managing CD include symptom alleviation, inflammation control, enhancement of quality of life, and mitigation of complication risks [5]. Despite advances, a definitive cure for CD eludes the medical community, necessitating a treatment regimen that often encompasses pharmacotherapy and surgical interventions. The heterogeneity in disease manifestation and therapeutic response necessitates the adoption of individualized treatment strategies, significantly elevating healthcare costs. Consequently, unraveling the molecular underpinnings of CD is imperative for the development of more efficacious and lower‐risk therapeutic modalities.

Colonic epithelial cells, integral to the intestinal barrier, facilitate nutrient and water absorption while impeding pathogen and toxin intrusion [6]. The inflammatory milieu endemic to CD detrimentally influences epithelial cell proliferation and differentiation. Inflammatory cytokines, such as tumor necrosis factor‐alpha (TNF‐α) and interleukin‐6 (IL‐6), can perturb the normal cell cycle, thereby impairing epithelial cell proliferation [7]. The oxidative stress and cellular damage engendered by chronic inflammation further compromise epithelial cell regenerative capacity [8]. The proliferation and migration of intestinal epithelial cells are critical for the restitution of the damaged intestinal barrier. In the aftermath of damage, adjacent healthy epithelial cells proliferate and migrate toward the damaged locale to re‐establish the barrier. Thus, attenuating inflammation to foster epithelial cell recovery and barrier repair emerges as a pivotal goal in CD therapy. Autophagy, a cellular self‐digestive process, sustains cellular homeostasis by degrading and recycling intracellular constituents [9]. In CD, dysfunctions in autophagy‐related genes impair the capacity to manage stress and excise damaged components, perpetuating chronic inflammation [10].

Circular RNAs (circRNAs), characterized by their closed‐loop structure devoid of 5' caps and 3' tails, emerge from the back‐splicing of precursor mRNAs, a process that eschews the linear sequence in favor of joining distant sites [11]. Recent investigations have underscored the role of circRNAs in modulating diverse biological processes in CD. hsa_circRNA_103124 has been shown to facilitate cell proliferation and block autophagy in CD by affecting the miR‐650/AKT2 signaling pathway [12]. Moreover, the increase in Hsa_circRNA_102610, serving as a molecular sponge for hsa‐miR‐130a‐3p, has been demonstrated to promote epithelial‐mesenchymal transition triggered by Transforming Growth Factor‐beta 1 [13]. hsa_circRNA_103124, by activating the AKT2 and Toll‐like Receptor 4/Nuclear Factor kappaB (NF‐κB) pathways, promotes M1‐type polarization of macrophages, sustaining the inflammatory milieu in CD [14]. While certain circRNAs have been identified for their biological significance in CD pathogenesis, the functions of myriad others remain to be elucidated. Therefore, further exploration into the roles of diverse circRNAs in CD pathogenesis is warranted. A profound understanding of the differential roles of circRNAs in CD will be instrumental in delineating the circRNA network in CD, potentially yielding more accurate biomarkers for diagnosis, prognostic assessment, and therapeutic efficacy monitoring in CD.

In the present study, through bioinformatic analyzes, we identified a circRNA, hsa_circ_0005255, that exhibits aberrant underexpression in CD and postulated that hsa_circ_0005255 plays a pivotal role in CD. This study is to explore the potential of hsa_circ_0005255 to act as a competitive endogenous RNA, sequestering miRNAs and thereby regulating the proliferation and autophagy of intestinal epithelial cells to ultimately restore intestinal barrier function.

## 2 Materials and Methods

### 2.1 Clinical Sample Collection

Informed consent was obtained from all participants. Between 2017 and 2021, 36 colonic specimens were collected from patients with CD undergoing elective colectomy at The First Affiliated Hospital of Anhui Medical University. Upon surgical removal, tissue samples were immediately cryopreserved in liquid nitrogen and subsequently stored at −80°C. Additionally, 23 normal colonic samples were collected from patients undergoing curative surgery for colorectal cancer, serving as controls. Comprehensive demographic information for all subjects is provided in Table S1.

### 2.2 Quantitative Reverse Transcription Polymerase Chain Reaction (RT‐qPCR)

Using Trizol reagent (Life Technologies), total RNA was collected from cells/tissues, and its quality was evaluated with a NanoDrop 2000 spectrophotometer (Thermo Fisher Scientific, USA). Using the PrimeScript RT Master Mix (Takara Bio, RR036A), reverse transcription was executed for circRNA and mRNA, and the PrimeScript TM RT Reagent Kit (Takara Bio, RR037A) was used for miRNA. The ChamQ SYBR qPCR Master Mix (Vazyme, Q311‐02) was utilized for conducting quantitative PCR on a Rotor Gene 3000 sequence detection system (Corbett Research, Australia). Gene expression was measured through the 2^−ΔΔCt^ technique and standardized in relation to GAPDH or U6. Primer sequences are detailed in Table 1.

**TABLE 1:** Primer sequences.

Gene	Primer sequences (5′–3′)
*Homo sapiens* circMAP4	Forward: 5′‐AGGGAGCGATACTACAGGCA‐3′
	Reverse: 5′‐TGGCAATGAAGTCCCGCTTT‐3′
*Mus musculus* circMAP4	Forward: 5′‐AACACTGCAGGCAGTCGCA‐3′
	Reverse: 5′‐TCCACAGTTTCTCCCACGATG‐3′
miR‐23a‐3p	Forward: 5′‐GCATCACATTGCCAGGG‐3′
	Reverse: 5′‐TGGTGTCGTGGAGTCG‐3′
NCOA3	Forward: 5′‐AGGATGCTTTCCAAGGCCAA‐3′
	Reverse: 5′‐TTAAGAAAACCCTGCTGGGAGC‐3′
U6	Forward: 5′‐CTCGCTTCGGCAGCACA‐3′
	Reverse: 5′‐AACGCTTCACGAATTTGCGT‐3′
*Homo sapiens* GAPDH	Forward: 5′‐CACCCACTCCTCCACCTTTG‐3′
	Reverse: 5′‐CCACCACCCTGTTGCTGTAG‐3′
*Mus musculus* GAPDH	Forward: 5′‐CATCAACGGGAAGCCCATC‐3′
	Reverse: 5′‐CTCGTGGTTCACACCCATC‐3′

### 2.3 Actinomycin D and RNase R Experiments

Caco‐2 cells were seeded in six‐well plates (5 × 10^5^ cells per well). Twenty‐four hours post‐seeding, cells were exposed to 2 μg/mL Actinomycin D (Sigma‐Aldrich) and harvested at specified time points for analysis. The stability of hsa_circ_0005255 and GAPDH mRNA was assessed via RT‐qPCR.

For RNase R treatment, RNA from Caco2 cells (10 μg) was incubated with RNase R (3 U/μg, Epicenter) at 37°C for 30 min. Subsequently, the stability of hsa_circ_0005255 and GAPDH was evaluated using RT‐qPCR.

### 2.4 Fluorescence In Situ Hybridization (FISH)

Subcellular localization of hsa_circ_0005255 in Caco‐2 cells was observed using a Cy3‐labeled hsa_circ_0005255 probe (Geneseed, Guangzhou, China). The Fluorescence In Situ Hybridization Kit (Geneseed, Guangzhou, China) was employed for the FISH analysis. The nuclei of cells were colored using 4',6‐diamidino‐2‐phenylindole (DAPI, Beyotime, China), and the photographs were taken with a fluorescence microscope (Leica, Wetzlar, Germany).

### 2.5 CD Mouse Model

Forty male C57BL/6J mice (8 weeks old, weighing 20–24 g) were acquired from Hunan SJA Laboratory Animal Co. Ltd. and housed in a controlled environment at 25°C ± 2°C with a 12 h light/dark cycle, with 1 week of acclimatization to the facility. Mice had ad libitum access to standard feed and clean water. To induce acute ulcerative colitis as a model for CD, 5% dextran sulfate sodium (DSS, Cayman Chemical Company) was dissolved in drinking water and administered for 5 days, followed by regular drinking water. For overexpression of hsa_circ_0005255, mice received a single intravenous injection of adenovirus‐associated virus (AAV) vector targeting hsa_circ_0005255 (0.2 mL, 10^12^ VP/mL) at 9 am on day 1. A similar volume of AAV negative control vector served as a control. The clinical course of the disease was monitored daily, evaluating weight loss, stool consistency, and rectal bleeding to calculate the Disease Activity Index (DAI). On day 8, euthanasia was performed via CO_2_ inhalation overdose, blood was collected by ocular extraction for serum separation, and the colon was excised, part of which was fixed in 4% paraformaldehyde and the remainder was stored at −80°C.

### 2.6 DAI Scoring

DAI was assessed daily based on weight loss, stool consistency, and the presence of blood in the feces. Weight loss was scored as follows: 0 points for an increase or decrease within 1% of baseline, 1 point for a decrease of 1%–5%, 2 points for 5%–10%, 3 points for 10%–15%, and 4 points for a decrease over 15%. Normal stool consistency was given 0 points, loose stools that do not adhere to the anus received 2 points, and liquid stools were assigned 4 points. A score of 0 was given for no blood in the feces, 2 for moderate bleeding, and 4 for severe bleeding.

### 2.7 Hematoxylin and Eosin (H&E) Staining

Colon tissues were collected from a 4% paraformaldehyde solution, dehydrated using ethanol, and then encased in paraffin. The embedded tissues underwent slicing at 5 μm, were deparaffinized using xylene, and sequentially rehydrated with ethanol. Following this, H&E staining took place, involving sections treated with a hematoxylin solution for 5 min, then differentiated using a hematoxylin solution and immersed in eosin. After dehydration, clearing, and mounting, the sections were examined using a microscope (BX 43 Olympus, Tokyo, Japan).

### 2.8 Terminal Deoxynucleotidyl Transferase dUTP Nick End Labeling (TUNEL) Assay

Apoptotic cells within colonic tissue specimens were identified using the TUNEL assay kit (C1088, Beyotime, China). Briefly, paraffin‐embedded sections were deparaffinized, rehydrated, and treated with proteinase K for 20 min. Samples were then incubated with a mixture of fluorescein‐labeled dUTP and Terminal deoxynucleotidyl Transferase enzyme in a humidified atmosphere at 37°C for 1 h. DNase I‐treated sections served as a positive control by incubating the section at room temperature (25°C) for 10 min before the fluorescence labeling procedure. Negative controls were incubated with dUTP alone at room temperature (25°C) for 10 min. Samples were subsequently treated with DAPI (Sigma‐Aldrich, Merck KGaA) for nuclear counterstaining, dehydrated through a graded ethanol series, cleared in xylene, and mounted with neutral resin. Images were acquired under a microscope (Carl Zeiss, LSM700).

### 2.9 Immunofluorescence (IF) Staining

For IF staining, sections of colonic tissue underwent deparaffinization using xylene, rehydration via gradient ethanol, followed by antigen retrieval and permeabilization with 0.3% Triton‐X 100. The samples were treated with Immunostaining Blocking Buffer (P0102, Beyotime, Shanghai, China) for blocking and then left to incubate with primary antibodies at 4°C overnight. The process involved incubating with secondary antibodies conjugated to fluorescence, such as goat anti‐mouse IgG Alexa Fluor Plus 488 (#A32723, Invitrogen) and donkey anti‐mouse IgG Alexa Fluor 594 (#A21203, Invitrogen). The nuclei underwent staining using DAPI (Sigma‐Aldrich), followed by analysis of the sections using a fluorescence microscope (BX 53 Olympus, Tokyo, Japan). This research utilized antibodies ZO‐1 (61‐7300, Invitrogen Antibodies) and F4/80 (ab6640, Abcam).

### 2.10 Enzyme‐Linked Immunosorbent Assay (ELISA)

Mouse blood was collected via ocular extraction and centrifuged at 4°C to obtain serum. Serum levels of TNF‐α, IL‐1β, IL‐6, and lipopolysaccharides (LPS) were quantified using ELISA kits. The ELISA kits for TNF‐α, IL‐1β, and IL‐6 were purchased from Thermo Fisher (Waltham, MA, USA), and the ELISA kit for LPS was acquired from Wuhan Jili De Biotechnology Co. Ltd. (Wuhan, China).

### 2.11 Western Blot Analysis

Total protein samples from cells and tissues underwent lysis with 500 μL of RIPA lysis buffer (Beyotime, China). Identical quantities of protein (20 μg) underwent separation using 10% SDS‐PAGE gels (Solarbio) and were then moved onto PVDF membranes (Invitrogen). The membranes underwent blocking using 5% skim milk for an hour, followed by an overnight incubation with primary antibodies at 4°C. The incubation with horseradish peroxidase‐linked goat anti‐rabbit secondary antibody IgG (1:1000, ab181236, Abcam) lasted for 2 h. The ECL reagent kit (34,080, Thermo Fisher Scientific) was utilized for detecting signals, while ImageJ software was employed for densitometric analysis. The primary antibodies employed were cleaved caspase‐3 (ab2302, Abcam), phospho‐p65 (p‐p65, 3031, Cell Signaling Technology), p65 (6956, Cell Signaling Technology), LC3B (2775, Cell Signaling Technology), p62 (5114, Cell Signaling Technology), and Beclin‐1 (3495, Cell Signaling Technology).

### 2.12 Cell Culture

The cell line utilized was the Caco‐2 cell line, which demonstrated the structural and functional traits typical of mature intestinal cells. Caco‐2 cells were placed in MEM (Cytiva HyClone, China), enriched with 20% fetal bovine serum (PAN‐Seratech, Germany), 1% sodium pyruvate, 1% non‐essential amino acids (Sigma, USA), 1% L‐glutamine, and a 100 U/mL penicillin–streptomycin blend (Gibco) at 37°C in an atmosphere containing 5% CO_2_. To induce an in vitro inflammatory model, Caco‐2 cells were stimulated with LPS (1 μg/mL, Sigma‐Aldrich, L2630) for 24 h.

### 2.13 Cell Transfection

Small interfering RNA (siRNA) for hsa_circ_0005255 and NCOA3, miR‐23a‐3p mimic/inhibitor, and their respective negative controls were purchased from Ribobio (Guangzhou, China). pcDNA 3.1 vectors overexpressing hsa_circ_0005255 and NCOA3 were acquired from HonorGene (Changsha, China). Transfection into Caco‐2 cells was performed using Lipofectamine 3000 (Thermo Fisher Scientific). Transfection efficiency was assessed 48 h post‐transfection using RT‐qPCR or western blot analysis.

### 2.14 Cell Counting Kit‐8 (CCK‐8) Assay

Caco‐2 cells were seeded in 96‐well plates (3 × 10^3^ cells per well) and cultured for a specified duration (0, 24, 48, and 72 h) before the addition of CCK‐8 solution (10 μL; Sigma). Absorbance at 450 nm was measured using a microplate reader (BIOTEK, Winooski, VT, USA) after incubating at 37°C for 1 h.

### 2.15 Transepithelial Electrical Resistance (TEER) Measurement

The intestinal barrier function of Caco‐2 monolayers was assessed by measuring TEER. Briefly, cells were grown on 0.36 cm^2^ and 0.4 μm pore polyester membrane transwell inserts in 12‐well plates at a density of 2 × 10^5^ cells/cm^2^. TEER measurements were taken using a dual electrode volt‐ohmmeter (Millicell‐ERS Resistance System, Millipore, Bedford, MA) at 37°C after treating cells with LPS or post‐transfection. The calculation for TEER is given by: TEER = (*R*_m_ − *R*_i_) × *A*, with *R*_m_ being the transmembrane resistance, *R*_i_ as the resistance of the cell‐free medium, and *A* as the membrane area in square centimeters.

### 2.16 Flow Cytometry

Apoptosis in Caco2 cells was quantified using an apoptosis detection kit (Beyotime). Cells were collected and incubated in the dark with 5 μL annexin V‐fluorescein isothiocyanate and 5 μL propidium iodide for 15 min. Cell apoptosis was assessed with a FACScan flow cytometer (Becton Dickinson, Mountain View, CA, USA).

### 2.17 Monodansylcadaverine (MDC) Staining

The cells were cultured overnight in 6‐well plates (2.5 × 10^5^ cells/well, 1 mL/well) and incubated with 50 μmol/L MDC at 37°C for 30 min. Following the incubation period, cells were rinsed twice with pre‐chilled PBS and captured under a fluorescence microscope (Invitrogen EVOS M7000, Germany).

### 2.18 Dual‐Luciferase Reporter Assay

The putative miR‐23a‐3p target sites within the 3'‐UTR of hsa_circ_0005255 and NCOA3 were PCR amplified from human genomic DNA and cloned into the pmiR‐RB‐REPORT vector (RiboBio), designated as hsa_circ_0005255‐WT and NCOA3‐WT. Mutant vectors, pmiR‐RB‐hsa_circ_0005255‐MUT and pmiR‐RB‐NCOA3‐MUT, were generated by mutating the miR‐23a‐3p target sites within the 3'‐UTRs using a PCR‐based method. Caco‐2 cells were co‐transfected with the above luciferase reporter vectors and miR‐23a‐3p mimic or mimic NC (GenePharma) using Lipofectamine 3000 (Invitrogen). Luciferase activity was measured 24 h post‐transfection using a dual‐luciferase reporter assay system (Promega, E2920).

### 2.19 RNA Immunoprecipitation (RIP) Assay

The RIP procedure was executed using the EZ‐Magna RIP Kit (Millipore). The lysis of Caco2 cells was carried out using a RIP lysis buffer. A similar amount of cell lysate (100 μL) underwent incubation with magnetic beads linked to mouse anti‐Argonaute 2 (Ago2) antibody in RIP buffer. Cellular extracts treated with standard mouse IgG were used as the negative control. Following this, RNA was measured using RT‐qPCR.

### 2.20 Data Analysis

Data were presented as mean ± standard deviation (SD). All experiments were replicated a minimum of three times to ensure biological repeatability. Statistical analysis was performed using GraphPad Prism version 9.0 (GraphPad Software, San Diego, CA, USA). Comparisons between two groups were analyzed using Student's *t*‐test, while comparisons among multiple groups were conducted using one‐way analysis of variance (ANOVA). Post hoc comparisons between groups were facilitated by Tukey's Honest Significant Difference (HSD) test. A *P value of < 0.05 was considered statistically significant.

## 3 Results

### 3.1 Differential Expression of hsa_circ_0005255: A Novel circRNA in CD Pathogenesis

**FIGURE 1:**
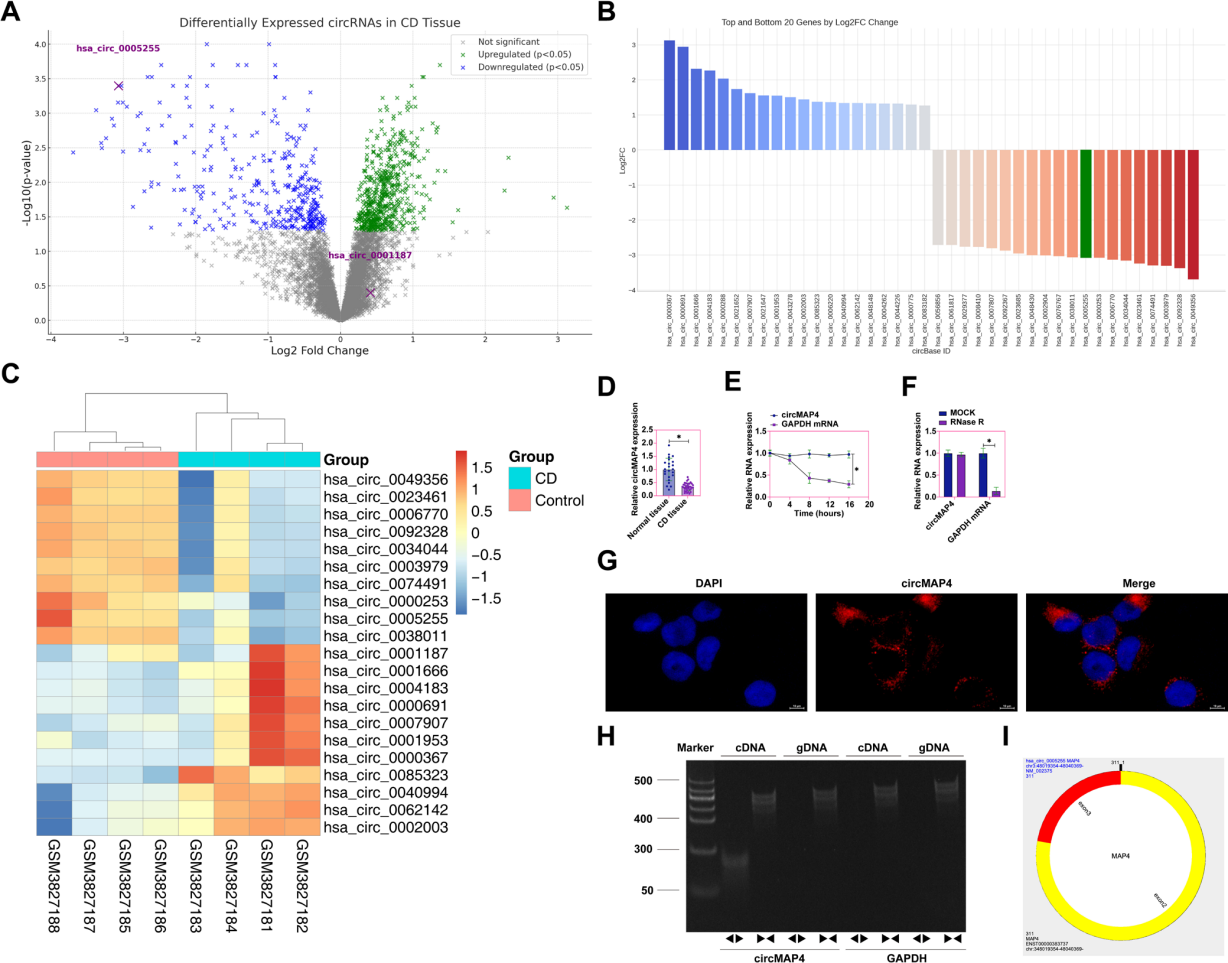
hsa_circ_0005255 is abnormally under‐expressed as a novel circRNA in CD pathogenesis. (A) Volcano plot showing the differential expression of circRNAs between normal colon tissues and those from patients with CD. The analysis revealed that 133 circRNAs were down‐regulated, and 47 circRNAs were up‐regulated in CD tissues compared to normal controls. (B) Bar graph depicting the top 20 most differentially expressed circRNAs in terms of fold change (Log2 scale) between normal and CD tissues. The bar graph reveals that the circRNAs with decreased expression (down‐regulated) exhibit higher fold changes than those with increased expression (up‐regulated). This observation emphasizes that CD pathogenesis is likely associated with a more substantial down‐regulation of certain circRNAs. (C) Heatmap illustrating the top 10 most up‐regulated and down‐regulated circRNAs in CD compared to normal tissues. The heatmap provides a visual representation of the differential expression patterns of these circRNAs, allowing for easy identification of their relative expression levels across the different samples. (D) RT‐qPCR analysis confirming the decreased expression of hsa_circ_0005255 in both normal and CD colon tissues. Normal tissue sample size was 23. CD tissue sample size was 36. (E) Actinomycin D, an inhibitor of RNA synthesis, was used to test the stability of circRNA. The lack of significant change in hsa_circ_0005255 expression suggests that its stability is independent of new RNA synthesis, potentially due to its circular structure, which is more resistant to degradation compared to linear RNA species. (F) RNase R digestion assay showing that hsa_circ_0005255 is resistant to RNase R digestion. RNase R is an exonuclease that selectively degrades linear RNAs, while circRNAs are known to be more stable due to their closed loop structure. (G) Fluorescence in situ hybridization (FISH) experiments demonstrating that hsa_circ_0005255 is predominantly localized in the cytoplasm of Caco2 cells. CircMAP4 was labeled with red fluorescence. DAPI was used to label the nucleus. (H) Specific primers for cDNA and gDNA were used to verify the presence of hsa_circ_0005255. This experiment rules out the possibility of genomic DNA contamination and confirms that the detected circRNA is a transcriptional product rather than genomic DNA. The specificity of the primers ensures the accuracy of the circRNA detection. (I) CircPrism software analysis revealing that hsa_circ_0005255 is derived from exons 2 and 3 of the MAP4 gene. The length of hsa_circ_0005255 is 311 bp. data are expressed as mean ± SD (*N* = 3).

In the quest to identify circRNAs implicated in the regulation of CD pathogenesis, our study utilized data from the GSE131911 database, comprising 4 healthy tissue samples and 4 CD tissue samples. Bioinformatic analysis conducted via R software revealed a differential expression pattern of circRNAs in CD, with 133 circRNAs being downregulated and 47 upregulated (Figure 1A), highlighting the potential pivotal role of circRNAs in the etiology of CD. Notably, among the top 20 differentially expressed circRNAs, those with decreased expression exhibited a generally higher fold change than those with increased expression. The fold change values (Log2 transformed) of these circRNAs were visually represented through bar graphs to elucidate the variation in expression patterns (Figure 1B). Heatmap analysis was also utilized to demonstrate the top 10 circRNAs with abnormally high and low expression (Figure 1C). Among these aberrantly expressed circRNAs, hsa_circ_0005255 was highlighted. hsa_circ_0005255 was found to be the only one of the top 10 abnormally expressed and underexpressed circRNAs with homologous genes in mice, which facilitated the study of its biological function in the CD mouse model. RT‐qPCR confirmed its reduced expression in both normal and CD‐affected colonic tissues (Figure 1D). Actinomycin D experiments showed that hsa_circ_0005255 has a special closed‐loop structure that allows it to exhibit high stability even under Actinomycin D treatment. Due to the lack of open ends, circular RNAs are more difficult to be degraded by exonucleases than linear RNAs, and thus their stability is less susceptible to transcriptional inhibition (Figure 1E). RNase R is an endonuclease that specifically degrades linear RNA but exhibits anti‐degradation of circRNA. In this study, the higher stability of hsa_circ_0005255 was further verified by the treatment of RNase R. Compared to linear RNA, hsa_circ_0005255 was not significantly degraded by RNase R treatment (Figure 1F). Subcellular localization studies via FISH demonstrated a predominant cytoplasmic accumulation of hsa_circ_0005255 in Caco2 cells, indicating its functional role within the cytoplasm (Figure 1G). The existence of hsa_circ_0005255 was conclusively validated through specific primers for cDNA and gDNA, effectively ruling out potential genomic DNA contamination (Figure 1H). Moreover, circPrism software analysis elucidated that hsa_circ_0005255 is composed of exons 2 and 3 of the MAP4 gene, forming a circular structure (Figure 1I). Collectively, these findings suggest that hsa_circ_0005255 is aberrantly underexpressed in CD, positing a potential regulatory role in the onset and progression of the disease.

### 3.2 Enhancement of Intestinal Epithelial Cell Proliferation and Autophagy in CD Mice via Overexpression of hsa_circ_0005255

**FIGURE 2:**
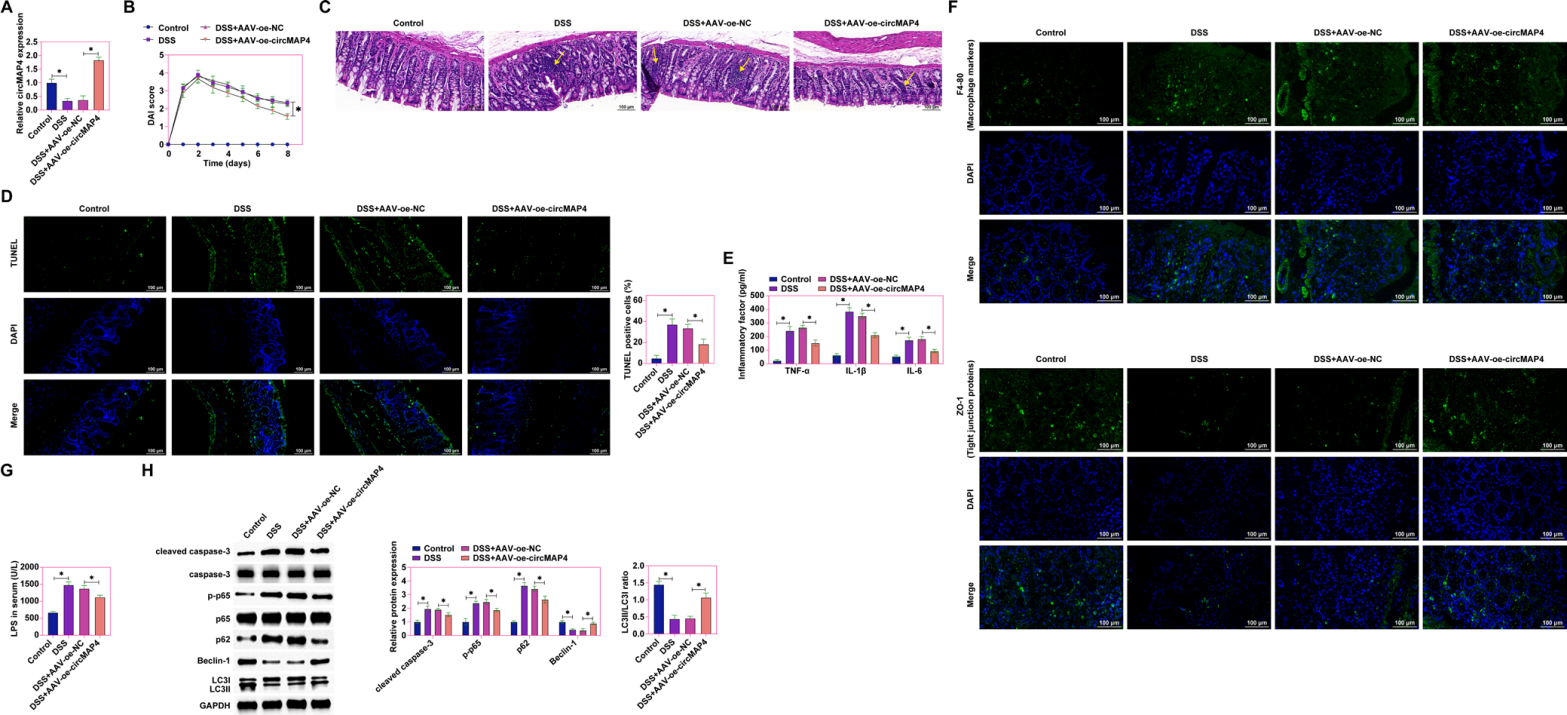
Overexpression of hsa_circ_0005255 significantly improves intestinal epithelial cell proliferation and autophagy in CD mice. CD mouse model was induced using DSS and AAV overexpression vector targeting hsa_circ_0005255 was injected into CD mice. (A) RT‐qPCR confirmed that hsa_circ_0005255 was lowly expressed in CD mice and was reversed by AAV‐oe‐hsa_circ_0005255; (B) DAI scores of each group of mice; (C) Representative images of HE staining of colonic tissues of mice in each group (Yellow arrows indicate inflammatory cell infiltration); (D) TUNEL staining to detect apoptosis in colonic tissues of mice in each group (green fluorescence to indicate TUNE positive cells and blue fluorescence for DAPI to label nuclei); (E) ELISA to detect the levels of inflammatory factors TNF‐α, IL‐1β, and IL‐6 in the serum of mice in each group; (F) Immunofluorescence to detect the expression of ZO‐1 and F4/80 in the colonic tissues of mice in each group (F4/80 is a macrophage marker. zo‐1 is a tight junction protein marker); (G) ELISA to detect the serum levels of LPS; (H) Western blot experiments to detect the expression of cleaved caspase‐3, p‐p65, LC3B, p62, and Beclin‐1 in colon tissues of mice in each group; data are expressed as mean ± SD (*N* = 8).

Subsequent investigations utilizing the DSS‐induced CD mouse model delineated the functional impact of hsa_circ_0005255 overexpression on disease pathogenesis. Initial validation via RT‐qPCR confirmed the underexpression of hsa_circ_0005255 in the CD model, with a significant upregulation observed post‐treatment with AAV‐oe‐hsa_circ_0005255 (Figure 2A). DAI assessments indicated elevated scores in CD mice, which were notably decreased upon hsa_circ_0005255 overexpression (Figure 2B). Histopathological analysis of colonic tissues revealed pronounced inflammatory cell infiltration in CD mice, a phenomenon markedly mitigated by hsa_circ_0005255 overexpression, thus ameliorating colonic architectural integrity (Figure 2C). TUNEL staining further illustrated a reduction in apoptosis rates within colonic epithelial cells following hsa_circ_0005255 overexpression, as evidenced by decreased TUNEL‐positive cells (Figure 2D). Inflammatory cytokine levels, quantified via ELISA, demonstrated significant elevations in TNF‐α, IL‐1β, and IL‐6 in the serum of CD mice, which were ameliorated following hsa_circ_0005255 overexpression (Figure 2E). Changes in F4/80, a marker of mouse macrophages involved in the intestinal immune response [15], and ZO‐1, a tight junction protein responsible for maintaining tight junctions in the intestinal epithelium [16], are capable of reflecting the integrity of the intestinal barrier. The barrier function of colonic epithelial cells was assessed, revealing that hsa_circ_0005255 overexpression significantly enhanced ZO‐1 expression and reduced F4/80 expression in CD mouse colonic tissues, as determined by immunofluorescence (Figure 2F). When intestinal barrier function is impaired, especially in CD patients, intestinal permeability is increased and LPS can enter the circulation from the intestinal lumen, triggering a systemic inflammatory response [17, 18]. This overexpression also led to a suppression of serum LPS levels, suggesting an enhancement in intestinal barrier function (Figure 2G). Cleaved caspase‐3 is a key marker in apoptosis, and its activation usually indicates that cells are undergoing programmed death [19]. p‐p65 is a marker of activation of the NF‐κB pathway, which is closely associated with inflammatory responses [20]. p62 is an important receptor in autophagy that binds to damaged proteins within the cell for removal [21]. LC3II and Beclin‐1 are important proteins related to autophagy, and an increase in LC3II is usually associated with the activation of autophagy, whereas Beclin‐1 is a key factor in the initiation of autophagy [22, 23]. Western blot analysis of apoptotic, inflammatory, and autophagy‐related proteins indicated that overexpression of hsa_circ_0005255 notably reduced the expression of cleaved caspase‐3, phospho‐p65, and p62, while increasing LC3II and Beclin‐1 expression in CD mouse colonic tissues (Figure 2H). Collectively, these findings elucidate that overexpression of hsa_circ_0005255 significantly enhances intestinal barrier functionality, autophagy, and attenuates inflammation in CD mice, highlighting its therapeutic potential in mitigating CD pathogenesis.

### 3.3 Amelioration of LPS‐Induced Intestinal Epithelial Cell Damage by Overexpression of hsa_circ_0005255 in Vitro

**FIGURE 3:**
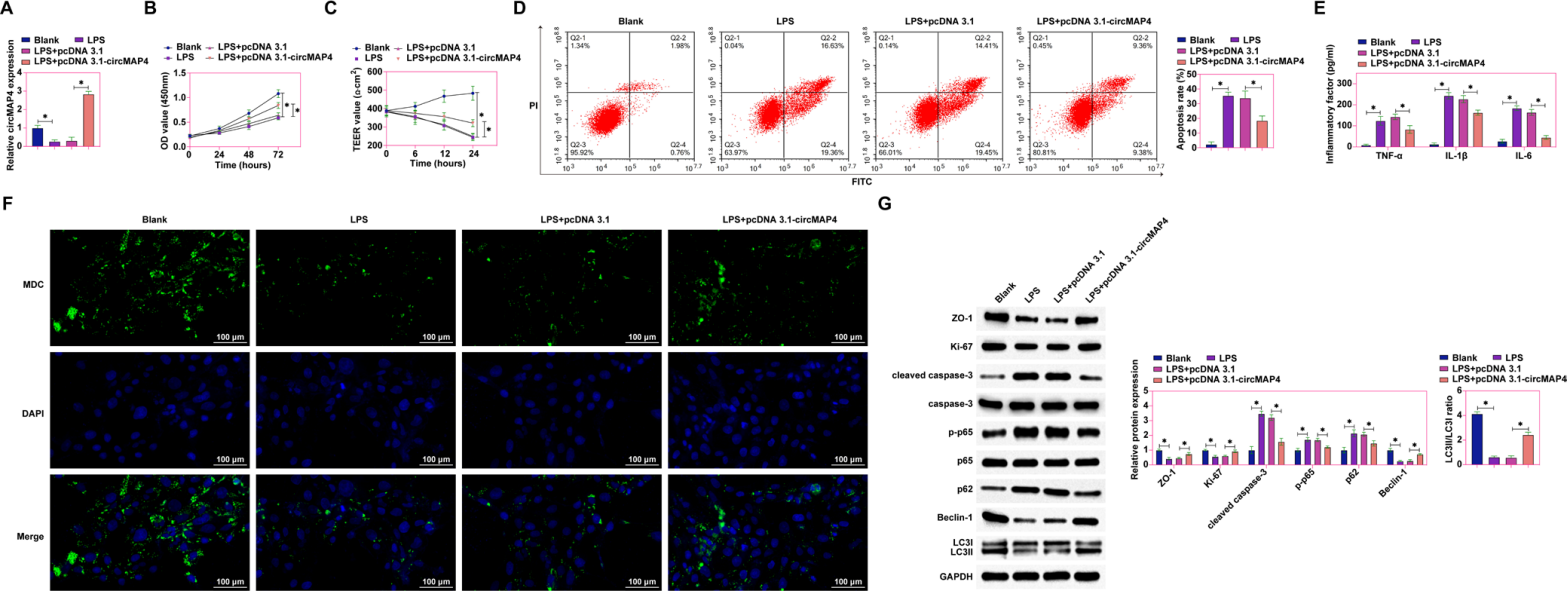
Overexpression of hsa_circ_0005255 ameliorates LPS‐induced intestinal epithelial cell injury in vitro. Caco2 cells were treated with LPS to establish an in vitro model of CD, and pcDNA 3.1‐hsa_circ_0005255 was transfected into Caco2 cells. (A) RT‐qPCR to detect the expression of hsa_circ_0005255; (B): CCK‐8 assay to detect the proliferation rate; (C) TEER test to assess the tight junction function of cells; (D) Flow cytometry to detect the apoptosis rate of cells; (E) ELISA kits to detect the levels of inflammatory factors TNF‐α, IL‐1β and IL‐6 in cells; (F) MDC staining to detect the formation of autophagosomes in cells; (G) Western blot assay to detect the expression of ZO‐1, Ki‐67, cleaved caspase‐3, p‐p65, LC3B, p62, and Beclin‐1 expression in cells; data are expressed as mean ± SD (*N* = 3).

To elucidate the protective effects of hsa_circ_0005255 against LPS‐induced damage in intestinal epithelial cells, an in vitro model of CD was established using LPS‐treated Caco2 cells, with subsequent overexpression of hsa_circ_0005255 facilitated by the pcDNA 3.1 vector. RT‐qPCR analyzes demonstrated a significant downregulation of hsa_circ_0005255 post‐LPS treatment, which was effectively reversed upon introduction of pcDNA 3.1‐hsa_circ_0005255 (Figure 3A). CCK‐8 assays revealed that LPS exposure reduced the proliferation rate of Caco2 cells, a deleterious effect significantly mitigated by hsa_circ_0005255 overexpression (Figure 3B). TEER measurements further indicated a diminution in barrier integrity in LPS‐treated Caco2 cells, an effect robustly counteracted by hsa_circ_0005255 overexpression, underscoring its crucial role in maintaining intestinal barrier function (Figure 3C). Flow cytometric analysis showed a notable reduction in apoptosis rates among cells overexpressing hsa_circ_0005255 compared to the LPS‐treated group (Figure 3D). Furthermore, ELISA results highlighted that overexpression of hsa_circ_0005255 effectively decreased the levels of pro‐inflammatory cytokines TNF‐α, IL‐1β, and IL‐6 (Figure 3E). MDC staining, employed to assess autophagosome formation in Caco2 cells, indicated a significant decrease in autophagosome numbers following LPS treatment, a phenomenon effectively reversed by hsa_circ_0005255 overexpression (Figure 3F). Ki‐67 is a classical marker of cell proliferation and is widely used to assess active division during the cell cycle [24]. Western blot analyzes provided insights into the expression patterns of tight junction, proliferation, apoptosis, inflammation, and autophagy‐related proteins. The results demonstrated that LPS exposure led to decreased expression of ZO‐1, Ki‐67, LC3II, and Beclin‐1, alongside increased expression of cleaved caspase‐3, phospho‐p65, and p62 in Caco2 cells. Notably, these alterations were prevented by overexpression of hsa_circ_0005255 (Figure 3G). Collectively, these data substantiate that overexpression of hsa_circ_0005255 efficaciously ameliorates LPS‐induced disruptions in intestinal epithelial cell barrier function, inflammation, and autophagy.

### 3.4 Competitive Sponging of miR‐23a‐3p by hsa_circ_0005255 Elucidates a Mechanistic Insight Into CD Pathogenesis

**FIGURE 4:**
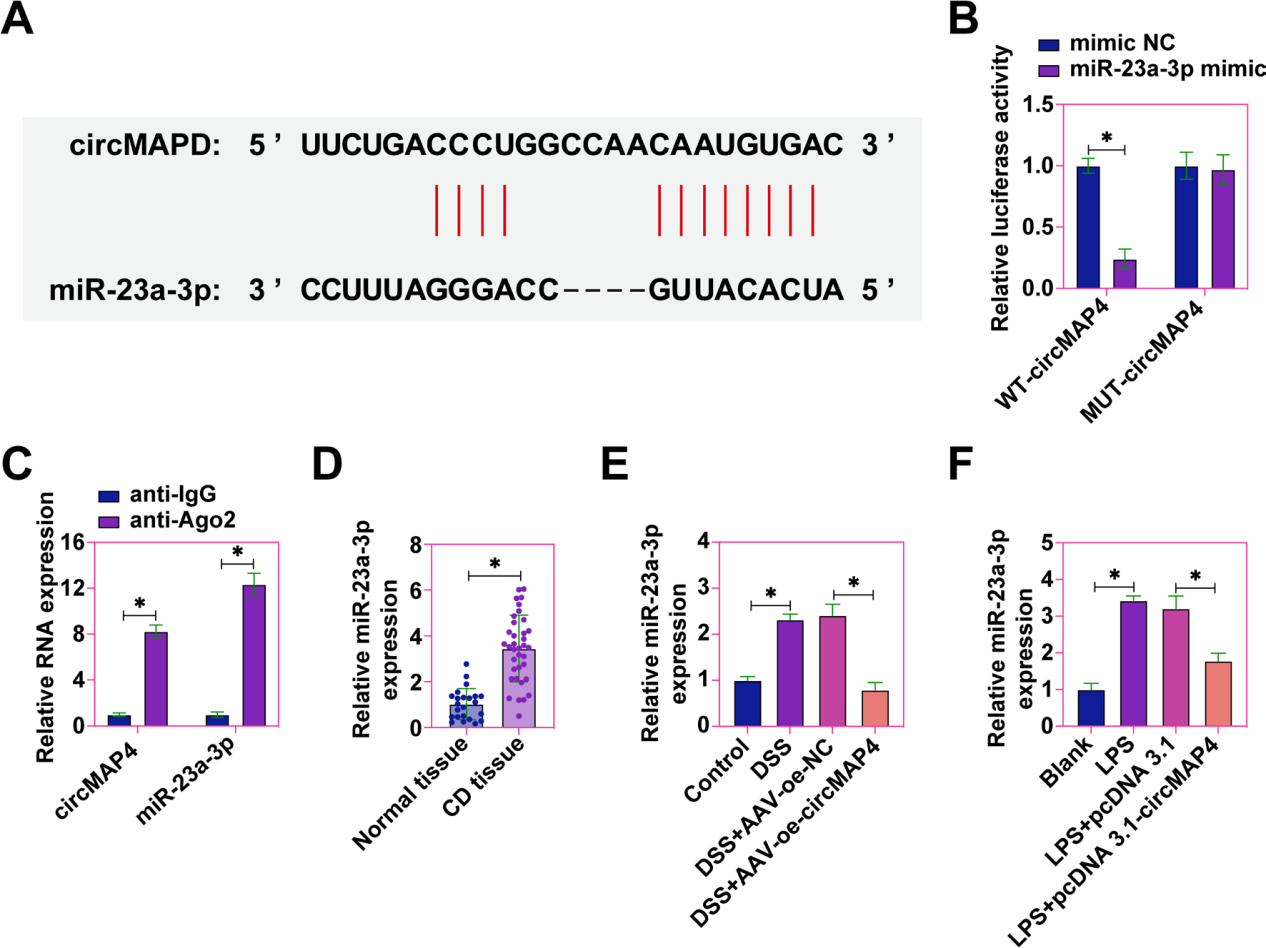
hsa_circ_0005255 competitively adsorbs miR‐23a‐3p. (A) Bioinformatics website starbase to predict potential binding sites for circNFIC and miR‐143‐3p; (B) Dual luciferase reporter assay to detect targeting relationship between hsa_circ_0005255 and miR‐23a‐3p; (C) RIP assay to detect the enrichment of hsa_circ_0005255 and miR‐23a‐3p in Ago2 magnetic beads; (D) RT‐qPCR to assess the expression of miR‐23a‐3p in colon tissues of CD patients and normal subjects; (E) RT‐qPCR to detect the expression of miR‐23a‐3p in colon tissues of CD mice; F: RT‐qPCR to detect the expression of miR‐23a‐3p in Caco2 cells; the data were expressed as mean ± SD (*N* = 3).

In the mechanistic elucidation of hsa_circ_0005255 functions, our interest gravitated toward the exploration of miRNAs potentially associated with and mediating its biological effects. Predicated on this premise, miR‐23a‐3p was selected for in‐depth investigation. Utilizing bioinformatics approaches, we identified potential binding sites between hsa_circ_0005255 and miR‐23a‐3p (Figure 4A). Dual‐luciferase reporter assays demonstrated a decrease in luciferase activity upon co‐transfection with WT‐hsa_circ_0005255 and miR‐23a‐3p mimic, whereas co‐transfection with MUT‐hsa_circ_0005255 and miR‐23a‐3p mimic did not affect luciferase activity (Figure 4B). Further validation through RIP assays showed significant enrichment of both hsa_circ_0005255 and miR‐23a‐3p in Ago2 complexes, confirming a targeted interaction (Figure 4C). Subsequent analyzes were directed at delineating the expression pattern of miR‐23a‐3p within the context of CD. Contrary to the underexpression of hsa_circ_0005255, miR‐23a‐3p exhibited aberrant overexpression in colonic tissues of CD patients (Figure 4D). In CD mouse models, miR‐23a‐3p expression was significantly augmented, whereas upregulation of hsa_circ_0005255 effectively mitigated the levels of miR‐23a‐3p in CD mice (Figure 4E). Additionally, LPS treatment induced an increase in miR‐23a‐3p expression in Caco2 cells, which was significantly suppressed upon overexpression of hsa_circ_0005255 (Figure 4F). These findings collectively underscore the role of hsa_circ_0005255 in competitively sponging miR‐23a‐3p.

### 3.5 hsa_circ_0005255 Mitigates LPS‐Induced Intestinal Epithelial Cell Damage by Modulating miR‐23a‐3p

**FIGURE 5:**
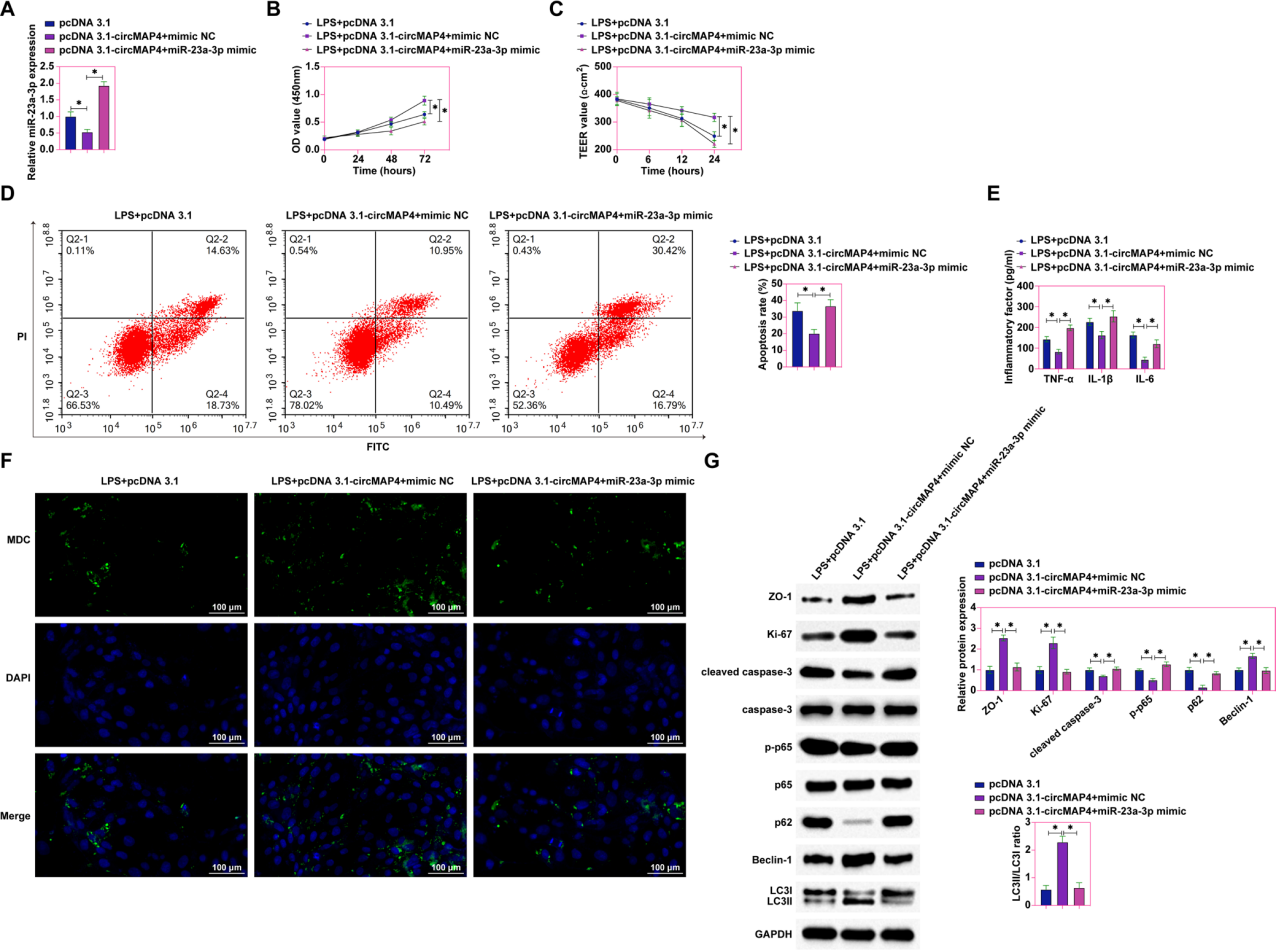
hsa_circ_0005255 ameliorates LPS‐induced intestinal epithelial cell injury in vitro by modulating miR‐23a‐3p. Caco2 cells were treated with LPS to establish an in vitro model of CD, and pcDNA 3.1‐hsa_circ_0005255 and miR‐23a‐3p mimic were co‐transfected into Caco2 cells. (A) RT‐qPCR to detect the expression of miR‐23a‐3p in cells; (B) CCK‐8 assay to detect the proliferation rate of cells; (C) TEER test to assess the tight junction function of cells; (D) Flow cytometry to detect the apoptosis rate of cells; (E) ELISA kits to assess the levels of inflammatory factors TNF‐α, IL‐1β and IL‐6 of cells; (F) MDC staining to detect the formation of autophagosomes of cells (green fluorescence indicates autophagic vesicles, blue fluorescence indicates nuclei); (G) Western blot assay to detect the expression of ZO‐1, Ki‐67, cleaved caspase‐3, p‐p65, LC3B, p62 and Beclin‐1 expression in cells; data are expressed as mean ± SD (*N* = 3).

To further elucidate the mechanism by which hsa_circ_0005255 ameliorates LPS‐induced damage in intestinal epithelial cells, we explored its regulatory relationship with miR‐23a‐3p. Co‐transfection experiments in LPS‐treated Caco‐2 cells were performed using pcDNA 3.1‐hsa_circ_0005255 and miR‐23a‐3p mimics. RT‐qPCR results indicated that the suppressive effect of pcDNA 3.1‐hsa_circ_0005255 on miR‐23a‐3p levels was reversed by co‐transfection with miR‐23a‐3p mimic (Figure 5A). CCK‐8 assays demonstrated that overexpression of hsa_circ_0005255 significantly increased the proliferation rate of Caco‐2 cells, an effect that was negated by the overexpression of miR‐23a‐3p (Figure 5B). TEER measurements further confirmed that hsa_circ_0005255 overexpression enhanced the TEER values, indicative of improved barrier integrity, which was reversed upon overexpression of miR‐23a‐3p (Figure 5C). Flow cytometric analysis revealed that the anti‐apoptotic effect exerted by hsa_circ_0005255 overexpression was countered by miR‐23a‐3p overexpression (Figure 5D). Moreover, ELISA assays showed that overexpression of hsa_circ_0005255 effectively reduced the levels of inflammatory cytokines TNF‐α, IL‐1β, and IL‐6, whereas these reductions were reversed with miR‐23a‐3p overexpression (Figure 5E). MDC staining, used to assess autophagic vesicle formation, indicated that hsa_circ_0005255 overexpression increased the number of autophagosomes, a phenomenon decreased by miR‐23a‐3p overexpression (Figure 5F). Western blot analysis further assessed the expression of tight junction, proliferation, apoptosis, inflammation, and autophagy‐related proteins. The results showed that hsa_circ_0005255 overexpression significantly promoted the expression of ZO‐1, Ki‐67, LC3II, and Beclin‐1, and inhibited cleaved caspase‐3, phospho‐p65, and p62 in Caco‐2 cells. However, these effects were reversed upon miR‐23a‐3p overexpression (Figure 5G). These findings collectively underscore that hsa_circ_0005255 exerts a protective role against LPS‐induced intestinal epithelial cell barrier damage, inflammation, and autophagy disruption by modulating miR‐23a‐3p.

### 3.6 NCOA3 Identified as a Downstream Target Gene of miR‐23a‐3p

**FIGURE 6:**
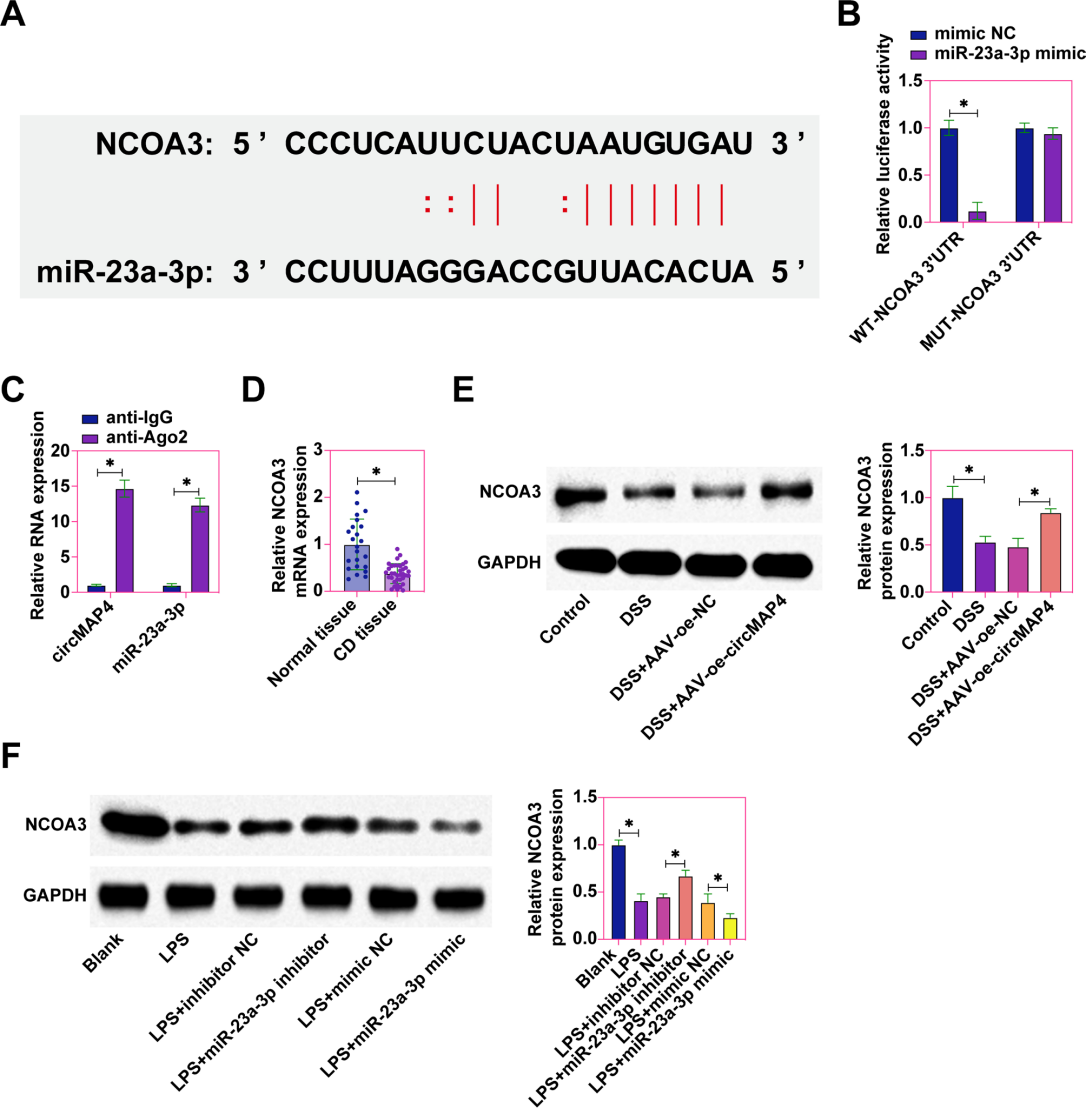
NCOA3 is a downstream target gene of miR‐23a‐3p. (A) Bioinformatics website starbase to predict the potential binding sites of NCOA3 and miR‐23a‐3p; (B) Targeting relationship of NCOA3 and miR‐23a‐3p detected by dual luciferase reporter assay in Caco2 cells; (C) Enrichment of NCOA3 and miR‐23a‐3p in Ago2 magnetic beads detected by RIP assay in Caco2 cells; (D) RT‐qPCR to assess the expression of NCOA3 in the colonic tissues of CD patients and normal subjects' colon tissues; (E) Western blot to detect the expression of NCOA3 in the colon tissues of CD mice; (F) Western blot to detect the expression of NCOA3 in Caco2 cells; data are expressed as mean ± SD (*N* = 3).

NCOA3 is a member of the nuclear receptor coactivator family. It plays a transcriptional regulatory role in the cell and is able to regulate the transcriptional level of genes by interacting with a variety of transcription factors and nuclear receptors [25]. Investigating the downstream molecular targets of miR‐23a‐3p, bioinformatic analyzes identified NCOA3 as a potential binding partner for miR‐23a‐3p (Figure 6A). To further characterize their targeting relationship, dual luciferase reporter assay and RIP assay were performed in Caco2 cells. As shown in Figure 6B, co‐transfection of WT‐NCOA3 and miR‐23a‐3p mimic significantly decreased luciferase activity in Caco2 cells, but co‐transfection of MUT‐NCOA3 and miR‐23a‐3p mimic did not affect luciferase activity in Caco2 cells. In addition, NCOA3 and miR‐23a‐3p were highly enriched in Ago2 magnetic beads compared with IgG controls (Figure 6C). Expression levels of NCOA3 were subsequently examined in the context of CD. In colonic tissues from CD patients, NCOA3 expression was found to be significantly lower compared to healthy control samples (Figure 6D). Similarly, in a CD mouse model, NCOA3 expression was reduced compared to controls, and overexpression of hsa_circ_0005255 effectively upregulated NCOA3 expression (Figure 6E). In vitro models further validated these findings, where LPS treatment significantly decreased NCOA3 expression in Caco2 cells, an effect that was exacerbated by miR‐23a‐3p overexpression but could be reversed by miR‐23a‐3p inhibitor treatment (Figure 6F). These findings collectively affirm NCOA3 as a downstream target of miR‐23a‐3p.

### 3.7 hsc_circ_0005255 Mediates NCOA3 to Attenuate LPS‐Elicited Intestinal Epithelial Damage

**FIGURE 7:**
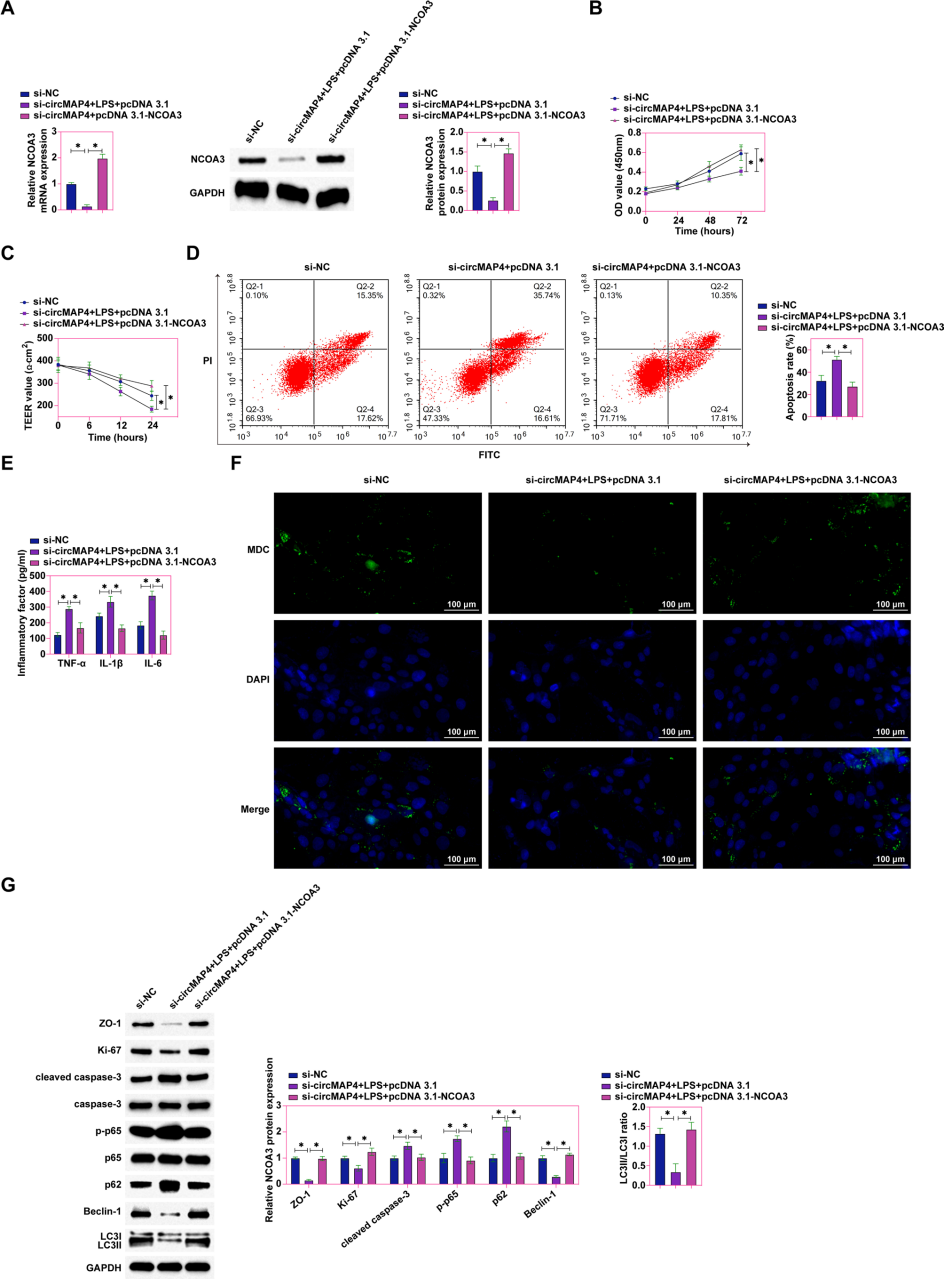
hsa_circ_0005255 ameliorates LPS‐induced intestinal epithelial cell injury in vitro by modulating NCOA3. Caco2 cells were treated with LPS to establish an in vitro model of CD, and si‐hsa_circ_0005255 and pcDNA 3.1‐NCOA3 were co‐transfected into Caco2 cells. (A) RT‐qPCR and western blot to detect the expression of NCOA3 and hsa_circ_0005255 in ells; (B) CCK‐8 assay to detect the proliferation rate of cells; (C) TEER test to assess the tight junction function of cells; (D) Flow cytometry to detect the apoptosis rate of cells; (E) ELISA kits to assess the levels of inflammatory factors TNF‐α, IL‐1β, and IL‐6 in cells; (F) MDC staining to detect the formation of autophagosomes in cells (green fluorescence indicates autophagic vesicles, blue fluorescence indicates nuclei); (G) Western blot assay to detect the expression of ZO‐1, Ki‐67, cleaved caspase‐3, p‐p65, LC3B, p62 and Beclin‐1 in cells; data are expressed as mean ± SD (*N* = 3).

Caco2 cells were co‐transfected with si‐hsa_circ_0005255 and pcDNA 3.1‐NCOA3 to explore the remedial potential against LPS‐induced cellular impairment. RT‐qPCR and Western blot analyzes established that si‐hsa_circ_0005255 significantly downregulated the expression of hsa_circ_0005255 and NCOA3, while pcDNA 3.1‐NCOA3 selectively restored NCOA3 levels, leaving hsa_circ_0005255 expression unaltered (Figure 7A). The CCK‐8 assay underscored that hsa_circ_0005255 silencing exacerbated the proliferative decline of Caco2 cells induced by LPS, an effect robustly countered by NCOA3 overexpression (Figure 7B). Further assessments revealed that hsa_circ_0005255 knockdown precipitated a decrease in TEER values, signaling compromised cellular junction integrity. This detrimental outcome was notably reversed following NCOA3 overexpression (Figure 7C). Flow cytometry analysis depicted an elevation in apoptosis rates upon hsa_circ_0005255 silencing, a condition significantly ameliorated by NCOA3 overexpression (Figure 7D). ELISA results demonstrated an upsurge in inflammatory cytokines TNF‐α, IL‐1β, and IL‐6 levels upon hsa_circ_0005255 knockdown, a trend effectively inverted by NCOA3 overexpression (Figure 7E). Autophagy analysis, via MDC staining, indicated a reduction in autophagosome formation following hsa_circ_0005255 silencing, an effect that NCOA3 overexpression effectively mitigated (Figure 7F). Western blot outcomes further reinforced the protective role of NCOA3, showing significant restoration in the expression of ZO‐1, Ki‐67, LC3II, and Beclin‐1, alongside a reduction in cleaved caspase‐3, p‐p65, and p62 levels in cells where NCOA3 was overexpressed, counteracting the deleterious effects instigated by hsa_circ_0005255 knockdown (Figure 7G). The compilation of these findings posits a pivotal role for the hsa_circ_0005255/NCOA3 axis in bolstering intestinal epithelial resilience against LPS‐induced disruptions.

## 4 Discussion

The global incidence and prevalence of CD are on the rise, notably within industrialized nations where changes in lifestyle and environmental factors have positioned CD as a significant public health challenge [26]. Although some studies have underscored the pivotal role of circRNAs in modulating CD inflammation and barrier integrity, the underlying mechanisms of circRNAs in CD pathogenesis remain to be elucidated. In this study, we identified a novel circRNA, hsa_circ_0005255, which is notably downregulated in the pathogenesis of CD, mediating NCOA3 by sequestering miR‐23a‐3p. This interaction is crucial for ameliorating intestinal barrier damage, promoting enterocyte proliferation, and autophagy in CD.

We discovered that hsa_circ_0005255 resists digestion by RNase R in intestinal epithelial cells, owing to its closed‐loop structure, thereby exhibiting greater stability than linear RNAs. This stability renders it a suitable biomarker in both intra‐ and extracellular environments. Numerous studies have indicated the potential of various circRNAs as biomarkers in CD pathogenesis, such as circular RNA_103516 [27], due to their specificity and sensitivity toward CD. In this study, hsa_circ_0005255 demonstrated significant downregulation in in vivo and in vitro models, as well as in clinical samples, suggesting its potential as a circulating biomarker for CD. However, future studies should evaluate the expression of hsa_circ_0005255 in patient sera and validate it through ROC analysis. This study primarily focuses on intestinal barrier function. The downregulation of hsa_circ_0005255 may be closely related to the chronic inflammatory environment of CD. First, inflammatory factors (e.g., TNF‐α, IL‐6) may inhibit its transcription through epigenetic regulation, leading to reduced expression. Second, dysregulation of the gut microbiota may also affect circRNA expression by altering cellular metabolism or regulating circRNA synthesis through molecules such as short‐chain fatty acids [28, 29]. Thus, down‐regulation of hsa_circ_0005255 may be the result of a combination of factors, including inflammation, epigenetic regulation, gut microbial imbalance, and intracellular stress response. Previous research has shown that upregulation of circSMAD4 disrupts the barrier function of intestinal epithelial cells in IL‐10 knockout mice [30]. Conversely, we found that upregulation of hsa_circ_0005255 preserves the integrity of tight junctions in enterocytes, contributing to reduced circulating LPS levels and ameliorating systemic inflammation in CD patients. This distinct role of different circRNAs in CD underscores the complexity of molecular mechanisms underlying CD pathogenesis. Furthermore, we demonstrated that hsa_circ_0005255 promotes proliferation in intestinal epithelial cells. Chronic inflammation and oxidative damage in CD can impair intestinal barrier function, but the regenerative proliferation of enterocytes can repair this damage [31]. We also found that hsa_circ_0005255 effectively promotes autophagy in Caco2 cells under LPS influence. Overexpression of hsa_circ_0005255 increases autophagosome numbers and enhances the expression of LC3II and Beclin‐1 proteins. When autophagy in enterocytes is impaired, their capacity to process intracellular pathogens (e.g., intestinal bacteria) diminishes, activating inflammatory pathways [32]. We posit that hsa_circ_0005255‐induced autophagy under LPS treatment facilitates resistance to inflammation and indirectly promotes proliferation. Previous studies, such as hsa_circRNA_103124, have shown to promote Caco2 cell proliferation while inhibiting autophagy [12], not using LPS treatment. Notably, inducing excessive autophagy under normal conditions can lead to autophagic cell death, potentially explaining reduced cell proliferation observed in that study.

While hsa_circ_0005255 shows a clear downregulation in CD patients, with evidence suggesting its involvement in preserving intestinal barrier integrity and promoting autophagy and enterocyte proliferation, hsa_circ_0001187 (previously reported in CD) did not exhibit significant changes in expression in our dataset (*p* = 0.3975). This discrepancy may stem from various factors, including differences in patient cohorts, disease severity, or methodologies. Our data revealed that hsa_circ_0001187 did not demonstrate significant differential expression in the studied samples, which could be due to clinical heterogeneity or the specific stage of the disease in the cohort we analyzed. It is possible that hsa_circ_0001187 could be more prominently involved in other stages of CD pathogenesis or in specific subsets of patients, a hypothesis that requires further investigation in larger, more stratified cohorts. Interestingly, the biological roles of these two circRNAs might diverge in their functional impact on CD progression. hsa_circ_0005255 appears to contribute to CD protection through autophagy, which plays a critical role in maintaining cell homeostasis and reducing inflammation by clearing damaged proteins and pathogens. In contrast, although hsa_circ_0001187 has been linked to CD in previous studies, its expression did not correlate with the markers of autophagy or inflammation in our study, suggesting that hsa_circ_0001187 might influence other aspects of disease progression, such as immune cell recruitment or mucosal healing, which would require more specific models and functional assays to explore further.

The role of autophagy in CD pathogenesis is complex. While autophagy has been recognized for its protective function in clearing damaged cells and maintaining homeostasis, excessive or impaired autophagy can contribute to disease progression. In this study, the promotion of autophagy by hsa_circ_0005255 in LPS‐treated cells aligns with a protective role in counteracting inflammation and injury. This is in contrast to studies such as Yin et al. [12], which report that in Crohn's disease, cell proliferation is promoted while autophagy is inhibited. This difference could stem from the experimental conditions; Yin et al. studied Crohn's disease in a model where autophagy was suppressed, potentially exacerbating inflammation, whereas our model highlights the induction of autophagy as a reparative response to LPS‐induced injury. We hypothesize that the induction of autophagy by hsa_circ_0005255 is a finely tuned process that aids in the clearance of damaged organelles and pathogens, thus promoting a more favorable environment for cell proliferation and barrier repair. The beneficial effects of autophagy observed in our study are likely due to its protective role in reducing inflammation and maintaining epithelial cell integrity, which may explain the observed recovery from LPS‐induced injury. It is essential to note that autophagy's role in inflammatory conditions may depend on its degree of activation. While moderate autophagy activation is protective, excessive or dysfunctional autophagy can contribute to cell death, as observed in some models of inflammatory diseases. The protective effects of autophagy observed in our study could be attributed to the context‐specific regulation of autophagy by hsa_circ_0005255, which may ensure that autophagy remains balanced and does not proceed to autophagic cell death, as may have occurred in the findings by Yin et al. Further studies focusing on the regulation of autophagy levels in response to circRNA overexpression could provide more insight into the molecular mechanisms at play.

Subsequent investigations revealed the capacity of hsa_circ_0005255 to act as a competitive endogenous RNA. Dual‐luciferase reporter assays and RIP experiments confirmed miR‐23a‐3p as a potential downstream miRNA of hsa_circ_0005255. The protective role of hsa_circ_0005255 on intestinal barrier function, as well as its promotion of autophagy and proliferation, was reversed upon overexpression of miR‐23a‐3p. Previous studies have shown miR‐23a‐3p to possess higher specificity and sensitivity than C‐reactive protein in ulcerative colitis patients, abnormally upregulated in inflammatory environments [33], highlighting its pivotal role in ulcerative colitis. Our findings further confirm the involvement of miR‐23a‐3p in regulating the progression of ulcerative colitis. miR‐23a‐3p's abnormal upregulation in LPS‐treated Caco2 cells disrupts cell proliferation and autophagy, exacerbating inflammation. Interestingly, recent studies have demonstrated that miR‐23a‐3p can reduce LPS‐induced macrophage inflammation [34], suggesting diverse biological functions of miR‐23a‐3p in different cells, highly relevant to its target protein regulation.

Furthermore, we validated that miR‐23a‐3p targets NCOA3 expression in Caco2 cells. NCOA3, a nuclear receptor coactivator, participates in regulating gene expression related to cell proliferation, metabolism, and development through binding to nuclear receptors. Previous research has indicated that NCOA3 suppresses inflammation and mediates goblet cell differentiation in DSS‐induced ulcerative colitis [35]. Our study lends further support to this notion. We also found that NCOA3 significantly promotes proliferation and autophagy in intestinal epithelial cells, protecting the intestinal barrier function, which is vital for suppressing intestinal inflammation.

This study has limitations; we have only confirmed the positive role of hsa_circ_0005255 in DSS‐induced CD, with its role in other CD animal models (e.g., IL‐10 knockout models) remaining unclear. Moreover, the limited sample size and the considerable heterogeneity among CD patients may not fully represent all CD cases. Thus, the generalizability of our findings needs validation in larger cohorts. Although this study explores the mechanism by which hsa_circ_0005255 modulates NCOA3 through sequestration of miR‐23a‐3p, other potential mechanisms and regulatory networks of circRNA remain to be elucidated in future clinical and basic research.

This study identified the downregulation of hsa_circ_0005255 in CD and preliminarily revealed its role in ameliorating intestinal barrier damage, promoting enterocyte proliferation, and autophagy through the mediation of NCOA3 by sequestering miR‐23a‐3p. Future research should focus on the expression patterns of hsa_circ_0005255 in larger samples and its effects in other CD‐relevant cell types to confirm its potential as a therapeutic target for CD. In summary, our study not only enhances the understanding of the molecular mechanisms of CD but also provides critical data support for the improvement of clinical diagnosis, treatment strategies, and public health management.

## Ethics Statement

All procedures performed in this study involving human participants were in accordance with the ethical standards of the institutional and/or national research committee and with the 1964 Helsinki Declaration and its later amendments or comparable ethical standards. All subjects were approved by The First Affiliated Hospital of Anhui Medical University (No. 201605HF‐13). All animal experiments were complied with the ARRIVE guidelines and performed in accordance with the National Institutes of Health Guide for the Care and Use of Laboratory Animals. The experiments were approved by the Institutional Animal Care and Use Committee of The First Affiliated Hospital of Anhui Medical University (No. 201612HF‐07).

## Conflicts of Interest

The authors declare no conflicts of interest.

## Supporting information


**Table S1.** Demographic information on patients with Crohn’s disease and colon cancer.

## Data Availability

The data that support the findings of this study are available from the corresponding author upon reasonable request.
